# Experimental study on the probabilities of kinked arches and kinked arch locations in ore passes under the influences of multiple factors

**DOI:** 10.1038/s41598-023-42519-x

**Published:** 2023-09-16

**Authors:** Jiaoyang Ma, Dianen Wei, Qingsong Zhang

**Affiliations:** 1 North China University of Science and Technology, Hebei, 063210 China; 2 Beijing Jiaotong University, Beijing, 100044 China; 3 Northeastern University, Liaoning, 110819 China

**Keywords:** Mineralogy, Civil engineering

## Abstract

A smooth shaft production process is important to ensure the safe and efficient development of mineral resources. Due to the influence of ore rock and the mismatch between rock size and ore pass design, an arching phenomenon occurs frequently during actual ore pass release, stagnating production. Therefore, an ore pass release simulator with a freely variable ore pass angle and height is designed and used to conduct a four-factor, five-level orthogonal ore pass release experiment. An analysis is performed to determine the influence of four factors—ore particle size, ore moisture, ore pass inclination and slip height—on the probability of ore pass arching; from there, an ore pass arching probability equation is constructed and the characteristics of the ore pass arching location are clarified, effectively predicting the probability of ore pass arching and providing an effective basis for handling arching events within an applicable range. This analysis effectively predicts the probability of ore pass arch within the applicable range, and provides an effective basis for handling ore pass arch events.

## Introduction

Ore passes are important structures of mine skid brake systems; they are related to the normal operation of ore transport between stages in actual production. Usually, due to differences in the physical and mechanical properties, including the ore particle size and moisture in the ore pass, the skid release ore often affects the quality and the mobility of the released ore; this effect may appear as an ore pass arch phenomenon^1^. If the arch phenomenon occurs too many times, it endangers production safety; in serious cases, it may cause ore pass scrapping, resulting in large economic losses^2–4^.

Many scholars have studied the problem of ore pass arching. Qiao Dengpan et al. analyzed the effects of different release conditions on the flow velocity of the ore bulk at the release port with random medium theory, and the researchers determined the characteristics of the distribution of the ore bulk movement velocity at the exit port^5,6^. Mogi proposed a gravity flow model considering the variation in the stack density of ore and the formation of small local arches, and the researchers used the model to study the effect of the ore pass dip angle on the flow characteristics of the ore^7^. Tao Ganqiang et al. conducted a bulk flow performance test on crumbling ore rock using a marker particle method, and they analyzed the effects of various factors, such as the size of the release opening and the ore particle size, on the bulk flow performance^8,9^. Yang et al. showed that the normal force and contact time decreased during impact as the inclination angle of the ore pass increased; they used a high-speed force measurement and acquisition system combined with a laboratory ore pass model^10^. Ma Chi et al. clarified the deformation and damage mechanisms and the damage zoning of an inclined ore pass wall by studying the motion characteristics of the ore bulk in the inclined ore pass^11^. Wang et al. analyzed the effects of the large particle size ore particle content and humidity on the flow capacities of ore particles by fitting test data from different mining sites^12^. Liu Yanzhang et al. used a combination of numerical simulations and similar tests to propose that the fines content and ore moisture in a suitable range significantly reduce the main ore pass arching phenomenon^13^. Hui Zhang et al. found that a power function variation characteristic between the modulus of blockiness and the mobility of ore release was observed; when the blockiness increased to a certain value, the ore pass was clogged^14^. Liu Yongtao et al. constructed a back propagation (BP) neural network model for the predictive analysis of ore pass arching^15^. Vo et al. used velocity field and discontinuous stress field methods in their study on ore pass arching, and they noted the relationships between ore pass arching, ore cohesion and water content^16^. Hadjigeorgiou et al. investigated the effects of ore pass geometry, rock fragment shape and size distribution on ore pass material flow^17^. To date, many scholars have studied the problems related to ore pass arching, mostly in terms of ore properties or ore pass arching and other single factors. Few studies have been conducted on methods for preventing ore pass arching in various aspects and for matching the ore particle size with the ore pass design under the influences of ore rock properties; this discrepancy makes it difficult to provide accurate theoretical guidance on ore pass design and methods for preventing and precisely locating ore pass blockage.

At the same time, the construction height of the ore pass is increasing, and the high ore pass is widely used. For example, when open pit mining with high steps, the high ore pass is used to ore pass ore to the bottom of the open pit^18,19^; when the deep shaft is lifted, stage transport motorcars dump ore into the high ore pass, and ore pass ore to the feeder shelter^20,21^; when bottomless pillar segmental avalanches are adopted for mine mining, the stage height increases with mining equipment. When the bottomless pillar segmental chipping method is used in mining, the stage height increases with the increase of mining equipment, and the ore pass height increases as well^22^. In the research of high sluice construction, Wang Ming et al. studied impact airflow under different unloading conditions and revealed the relationship between impact airflow and unloading volume and unloading height^23^. Wang Jiuzhu et al. investigated differential pressure, airflow velocity and its influencing factors at the unloading port of a multi-center high sluice^24^. Jiang Zhong'an et al. revealed changes in airflow and dust transport laws in the ore pass^25^. Xiong Xiaochen combined mine examples to form high slipway construction standards and management methods, which accelerated the construction progress of high slipway^26^. The emergence of high sliding shafts is inevitable. The emergence of new things will bring new problems. The previous measures used to deal with the problem of slip shafts can not be well adapted to the problem of high slip shafts, so carrying out experimental research on the high slip shaft mine is extremely important, with a certain degree of foresight.

To prevent the occurrence of ore pass arching and accurately address ore pass arching events, an ore pass release simulator was developed to simulate the release environment and record the arching phenomenon. In addition, we carry out a statistical analysis of the experimental results, obtain information on the frequency and location of the arching phenomenon, explore the causes of the occurrence of ore pass arching, determine the probability of occurrence and uncover the characteristics of the arching location. This study may provide guidance and suggestions for the construction of high depth ore passes and the prevention and treatment of ore pass arching problems in mines. This study will provide guidance and suggestions for the construction of high depth ore passes and the prevention and treatment of ore pass arching.

## Similar simulation experiments of mine release from the ore pass

### Ore pass release simulator

Ore pass field release experiments experience difficulties in operation and obtaining data, and they are extremely dangerous and expensive. In contrast, similar experiments have a high operability factor, safety, and low cost, and data near the actual project are obtained by controlling the experimental conditions and by standardizing the experimental steps^27^; this method is effective for studying ore pass arching. As mining technology matures and large-scale equipment is produced, the stage height gradually increases, the skid hole height increases, and skid hole clogging and arching problems largely affect the continuity of mine mining^28,29^. Therefore, it is necessary to design and produce a physical model of mine release that can collect and analyses data on the ore clogging location and number of clogs during mine release based on the specific conditions of the process in the field.

**Figure 1:**
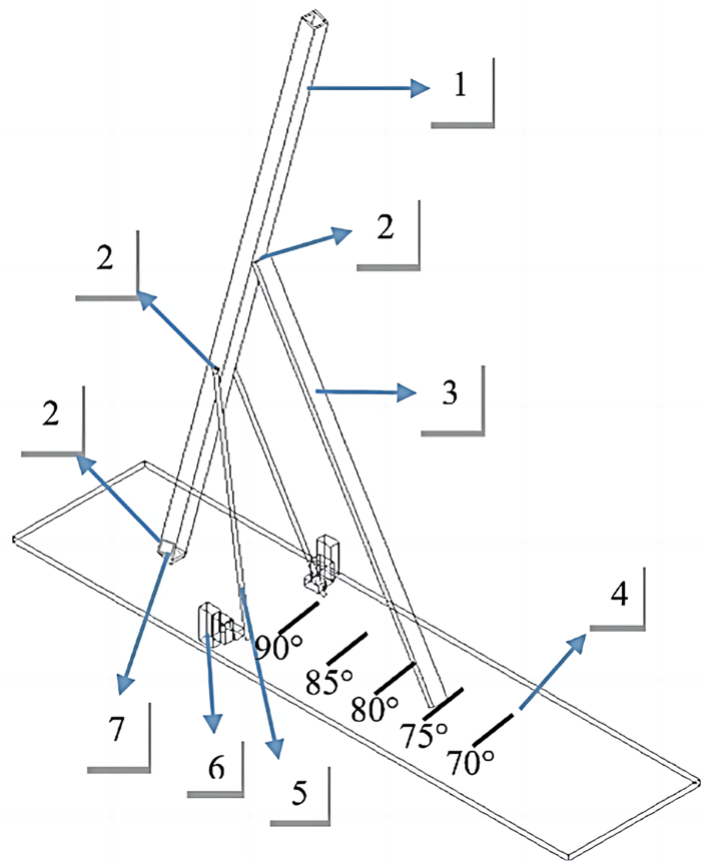
Principle diagram of the ore pass release simulator. 1—Skidhole pipe; 2—acrylic hinge connection; 3—bracket; 4—base; 5—both sides of the bracket; 6—bracket fixings; 7—release port.

Existing ore pass release simulation equipment is based on the specific conditions of the field ore pass release, and the equipment is prepped for an equal grain size arch experiment. Figure 1 shows the principle diagram of the ore pass release simulator. The height of the equipment is not adjustable and cannot simulate a high ore pass release simulation experiment. The ore pass release simulation equipment is 90°, and it cannot change the inclination angle. There are various problems, such as a small ore pass height, an unchangeable tilt angle, few adjustable factors within the experimental model, and overly targeted site conditions. Therefore, the acrylic tube height of the simulated ore pass selected for the ore pass release experimental device in this project is 160 cm; this tube can simulate a high inclination ore pass and any slip height below 160 m.

To make the angle of the ore pass simulator adjustable, an angle control method for the ore pass simulation tube is proposed. By cutting the slot at the bottom of the base, the base and ore pass simulation tube side uses a hinge for connection (the hinge is removable, making it easy to replace the ore pass simulation tube); the control of the ore pass tube experiences relative deflection, with the hinge being a wooden bracket at one end and the middle of the ore pass simulation tube being fixed. The other end of the base has a corresponding angle scale, and the bracket is used for angle control, thus making the inclination of the skid hole simulator freely changeable. The angle scale is inscribed with the cosine theorem of a triangle as the basis of the theory.

The side of the ore pass simulation tube deflection occurs with two brackets fixed. Due to the high height and weight values of the ore pass undergoing ore release, the easy destabilization during release, and the fixed iron support of the ore pass release simulator, the simulation of the ore pass acrylic tube constitutes a triangular cone structure and can guarantee the stability of the model when filled with ore. The whole triangular cone is stable when the three corners of the cone bottom provide a support force with the same direction of target and similar strength^30^. The inclination angle is calculated by the equation tan α = S/L(α denotes the angle of inclination of the ore pass, S denotes the vertical height from the connection of the bracket and the ore pass tube to the bottom of the base, and L denotes the distance between the point of vertical projection of the connection of the bracket and the ore pass tube to the base and the pivot point where the base and the ore pass tube are connected.), and the actual angle of the ore pass model is controlled by marking the scales on the bottom plate and the movable support frame; the actual height is marked at each position of the square tube and the slip height is controlled by controlling the amount of ore added during the experiment.

The ore pass release simulator is made of acrylic and consists of base, ore pass tube, bracket and angle control wooden pole. The parameters of each accessory are shown in Table 1. The dimensions of the ore pass simulator tube are H × L × W = 160 cm × 3 cm × 3 cm acrylic columnar hollow tube, the thickness of the acrylic material is 0.5 cm, the base is L × W × T = 150 cm × 50 cm × 5 cm acrylic plate, the thickness of the plate is 1 cm, the ore pass simulator tube is connected to the base with the use of hinges on one side and the base corresponds to the simulation tube of the rest of the three sides, and a slot is engraved at the simulation tube to prepare for the simulation of the ore pass inclination change with a certain degree of freedom. One side of the simulated tube is connected to the base with a hinge, and the other three sides of the simulated tube are engraved with slots to provide a certain degree of freedom when the simulated tube simulates changes in ore pass inclination. The ore pass simulation tubes are marked with 25 cm, 50 cm, 75 cm, 100 cm, 125 cm and 150 cm from the base. At the height of the sliding well simulation tube 50 cm on both sides, the use of hinges connected to the two iron brackets, the iron bracket on the other side of the square wood block fixed on the base, the wood block and the base using threaded connection, can be regularly mobilized, to play a fixed support role; the height of the sliding well simulation tube 100 cm, the use of acrylic hinges connected to the angle of control of the wooden rod, the base surface is marked with a tilt angle scale, to play a role in controlling the angle of inclination. The surface of the base is marked with the tilt angle scale number, playing the role of controlling the tilt angle.

**Table 1:** Accessories parameters of ore drawing simulator.

Structure	Material quality	Parameter
Skidhole pipe	Plexiglass	160 cm × 3 cm × 3 cm × 5 mm
Bracket	Timber	120 cm × 5 cm × 3 cm
Base	Plexiglass	150 cm × 40 cm × 1 cm
Both sides of the bracket	Iron	50 cm × 5 cm × 5 cm
Bracket fixings	Timber	10 cm × 5 mm × 5 mm
Acrylic hinge connection	Plexiglass	3 cm × 3 cm

### Determination of the natural angle of repose and the sliding friction angle of experimental ores

The action of forces is the fundamental reason for the occurrence of kinked arch in the ore pass. Bonding/adhesion, compaction, occlusion, etc. are the phenomena of their force action. Adhesion occurs when the residual force field on the surface of a solid and the mass of the solid or liquid in close contact with it are attracted to each other. The essence of the adhesion phenomenon and adsorption is the same. They are the result of surface forces between two substances. Adhesion can be shown by the friction of two solids sliding relative to each other, the aggregation of solid powder and other phenomena. Natural resting angle and sliding friction angle can be used as quantitative characterization parameters for adhesion phenomena. The internal friction angle reflects the friction characteristics between the layers of the bulk material, and the natural rest angle indicates the ability of the single grain material to roll down on the material pile, which is the appearance of the internal friction characteristics. Bonding generally refers to the bonding force. The natural angle of repose of bonded particles is consistent with the angle of internal friction^31^. The repose angle can qualitatively represent the mobility of the material; the smaller the repose angle, the better the mobility and the weaker the bonding^32^. That is, in this paper, the natural angle of repose and the friction angle are used to characterize the degree of bonding strength.

#### Natural resting angle

After the natural resting angle is formed, adding bulk material to the pile again, it will naturally slip down and maintain this angle, which will only increase, while increasing the bottom area. The natural resting angle of ore with different moisture content and particle size is shown in Table 2. With the increase of ore particle size, the natural resting angle shows a trend of increasing and then decreasing.

**Table 2:** Natural resting corner of the experimental ore.

Ore moisture						
Natural rest corner	0%	1%	1.5%	2%	2.5%	3%
Ore size						
0–2 mm	25.64°	34.61°	32.21°	38.31°	40.03°	25.64°
2–4 mm	33.02°	37.23°	36.13°	38.66°	42.61°	33.02°
4–6 mm	31.38°	34.61°	33.02°	40.03°	38.31°	31.38°
6–8 mm	30.96°	32.62°	31.80°	35.75°	34.99°	30.96°
8–10 mm	30.11°	31.80°	30.54°	35.37°	34.61°	30.11°

#### Sliding friction angle

The friction angle can be mechanically understood as the critical self-stabilizing angle of the block on the sloping surface, within which the block is stable. It can characterize the friction and sliding characteristics of the material with the container wall. The sliding friction angle reflects the friction characteristics and shear strength between bulk materials. The ore body as a whole, in any place inside it to take out a unit body, the unit body on the unit area of the normal pressure can be regarded as the surface of the compressive stress, the unit area of the shear force can be regarded as the surface of the shear stress. The ore mass slides towards the shear. That is, the flow of ore bulk can be seen as similar to the damage phenomenon of solid shear flow. The sliding friction angle of ore with different moisture content and particle size is shown in Table 3.

**Table 3:** Sliding friction angle of the experimental ore.

Ore moisture						
Sliding friction angle	0%	1%	1.5%	2%	2.5%	3%
Ore size						
0–2 mm	29.5°	36.5°	42.5°	44.5°	55°	58.5°
2–4 mm	31°	45.5°	47.5°	53°	57.5°	62.5°
4–6 mm	31°	44°	47°	47.5°	46.75°	52.5°
6–8 mm	28°	34.75°	41.5°	43.25°	43°	44.5°
8–10 mm	28°	33°	34.75°	35°	36.5°	38.5°

### Experimental program and implementation of slip ore release

Bulk rocks of different grain sizes were selected for sun drying and mesh sieving. In the experiment, the moisture requirement was achieved by spraying with a spray bottle according to the required ore moisture. Ore with particle size of 0–10 mm and humidity of 1–3% after wet screening and spraying was used as experimental raw material. Experimental factors are ore particle size, ore moisture, slip height, ore pass inclination. The factors are divided into five levels, the level range of ore particle size: 0–10 mm, ore moisture: 1–3%, slip height: 50–150 cm, ore pass inclination: 70°–90°. The orthogonal experimental analysis method, which is the most commonly used multi-factor experimental analysis method^33–35^, was proposed in conducting compatibility tests due to only four factors, while the L_25_(5^6^) standard orthogonal table can be arranged with up to six factors^36^. Consequently, the data in columns 5 and 6 need not be taken into account when designing test cases. The level of factor 4 for the orthogonal experiment was determined to be 5, i.e., L_25_(5^4^). The orthogonal experiment factor levels are shown in Table 4. The orthogonal experiment table is shown in Table 5.

**Table 4:** Factor levels for orthogonal experiments.

Level	Factor			
	Ore moisture (%)	Ore particle size (mm)	Ore pass inclination (°)	slip height/cm
1	1	0–2	90	50
2	1.5	2–4	85	75
3	2	4–6	80	100
4	2.5	6–8	75	125
5	3	8–10	70	150

**Table 5:** Orthogonal experiment table.

Experimental number	Ore moisture (%)	Ore particle size (mm)	Ore pass inclination (°)	slip height (cm)
1	1	0–2	90	50
2	1	2–4	85	75
3	1	4–6	80	100
4	1	6–8	75	125
5	1	8–10	70	150
6	1.5	0–2	85	100
7	1.5	2–4	80	125
8	1.5	4–6	75	150
9	1.5	6–8	70	50
10	1.5	8–10	90	75
11	2	0–2	80	150
12	2	2–4	75	50
13	2	4–6	70	75
14	2	6–8	90	100
15	2	8–10	85	125
16	2.5	0–2	75	75
17	2.5	2–4	70	100
18	2.5	4–6	90	125
19	2.5	6–8	85	150
20	2.5	8–10	80	50
21	3	0–2	70	125
22	3	2–4	90	150
23	3	4–6	85	50
24	3	6–8	80	75
25	3	8–10	75	100

In order to avoid the chance of ore pass release during the experiment, each of the 25 sets of experiments in the orthogonal table will also be performed 10 times, thus reducing the influence of small probability events on the experiment.

The experimental operation steps are: (1) setting the sluice inclination parameter of the sluice discharge simulator; (2) sieving the experimental ore, weighing the ore, and calculating the amount of water according to the formula(*x*/S_z_ + *x* = *b*, where *x* is the amount of water added, L; *b* is the percentage of moisture; S_z_ is the ore quality, kg); (3) mixing the water with the experimental ore evenly; (4) placing the experimental ore in the ore pass discharge simulator to reach the corresponding material level; (5) uniformly releasing the ore from the discharge opening, and recording the location of the arch if arching occurs; (6) performing the above 25 groups of experiments, each group of ten operations, and record the arching situation.

## Analysis of the probability of arch formation in the ore pass under the influences of various factors

### Analysis of the influences of single factors

#### Influence of ore moisture on the probability of ore pass arch

**Figure 2:**
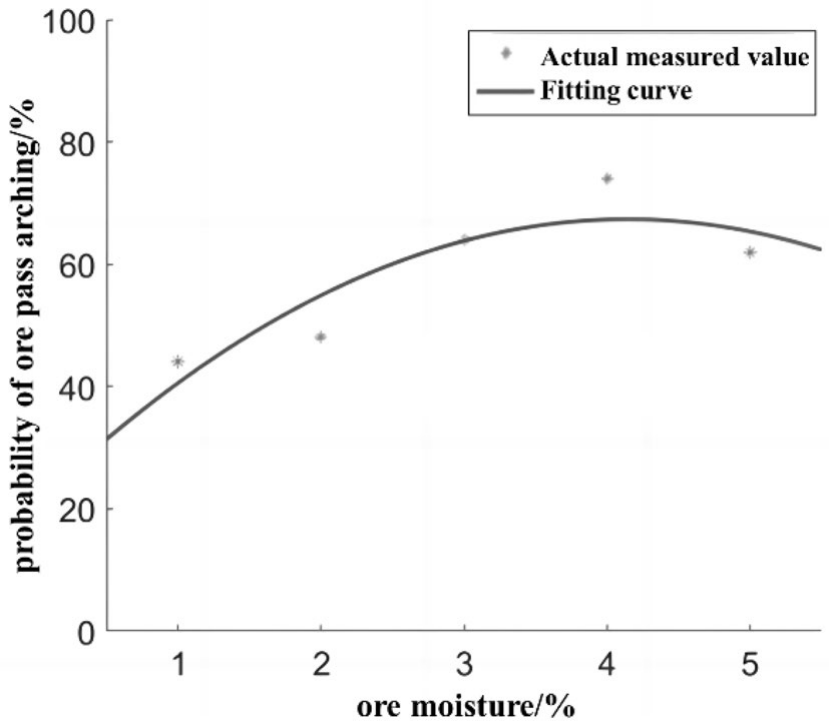
Effect of ore moisture on ore pass arch probability.

The probability of the ore pass under different ore moistures is fitted, and the fitted image of the influence of ore moisture on the probability of the ore pass arch is shown in Fig. 2.

The fitted equation for the probability of ore pass arching under the influence of ore moisture is shown in Eq. (1), and the fitted residual mode is 10.2, indicating a good fit. The applicable condition is that the ore moisture is in the range of 1–3%.

1 $$P_{ca} = - 2.7M_{cL}^{2} + 22M_{cL} + 21$$

where *P*_ca_ is the probability of ore pass arching, %; *M*_*cL*_ is the ore moisture, %.

Ore moisture affects the probability of the ore pass arch equation; the absolute value of the quadratic term coefficient is small, the influence of ore moisture on the probability of the ore pass arch is relatively low, and the ore moisture and probability of the ore pass arch have a quadratic relationship. Combined with similar simulation results and the ore sliding friction angle, when the ore moisture is in the range of 1–2.5%, moisture in the form of bound water creates water films on the surfaces of the ore particles between the ore and the wall of the ore pass to produce bonding; when the natural resting angle of the ore particles is the highest, the bond between the ore particles and the friction coefficient between the ore and the wall of the ore pass increase with the increase in ore moisture, making it easy for the ore to bond to the arch, thereby gradually increasing the probability of ore pass arching. When the ore humidity reaches 2–2.5%, the experimental bulk bonded water is used to reach saturation. Then, with the increase in the moisture content of the ore bonded water remaining unchanged, the excess water in the form of free water destroys the bonding effect of bonded water, the lubrication effect appears, and the probability of arch decreases.

**Figure 3:**
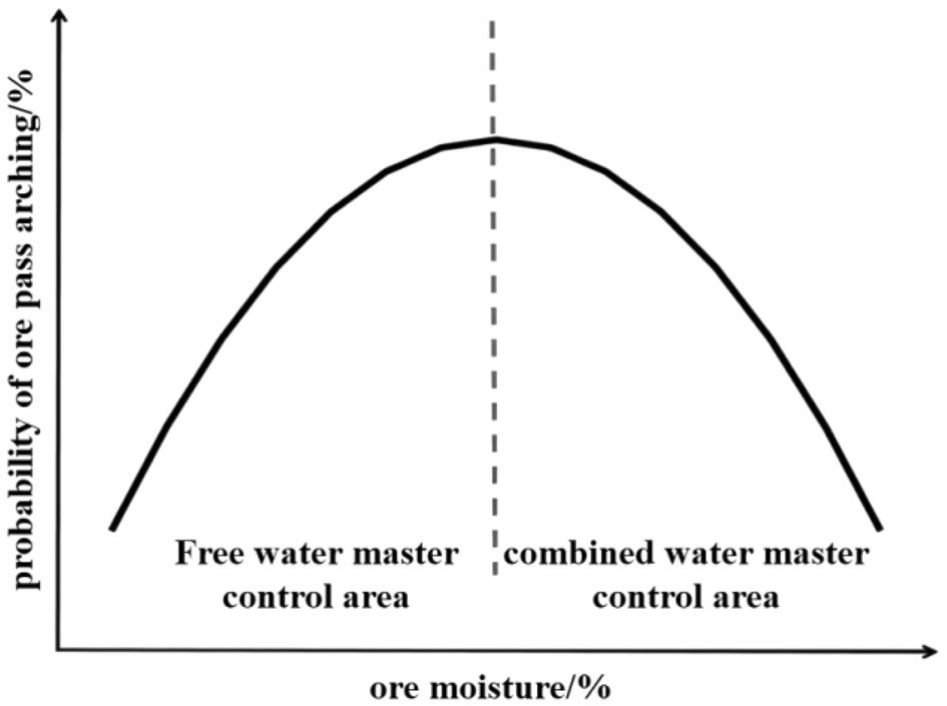
Correlation between ore moisture and the probability of arching in the ore pass.

In summary, before the ore moisture reaches the saturation water content, the bonded water controls the arching probability of the ore pass; after reaching the saturation water content, the free water controls the arching probability of the ore pass (Fig. 3).

#### Effect of ore particle size on the probability of ore pass arching

**Figure 4:**
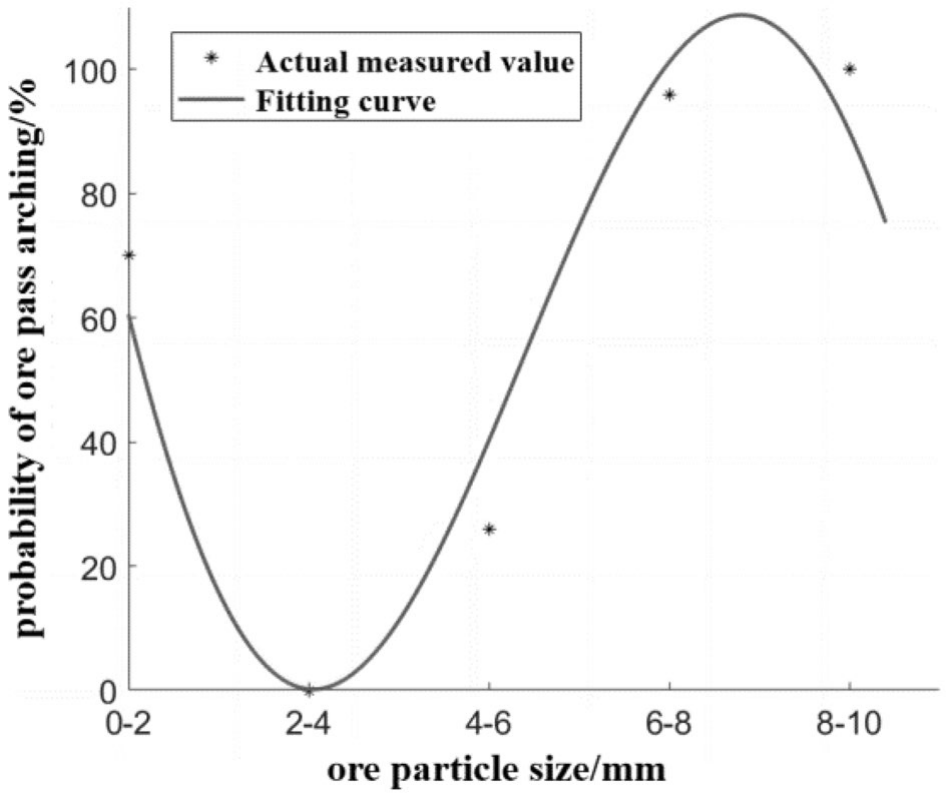
Effect of ore particle size on the probability of arch formation in the ore pass.

The probability of the ore pass under different ore particle size is fitted, and the fitted image of the influence of ore particle size on the probability of the ore pass arch is shown in Fig. 4.

Since R^2^ = 0.9547, the fitting effect is good; therefore, the influences of the ore particle size under the ore pass arch probability are fit with Eq. (2), which is applicable for ore particle sizes in the range of 0–10 mm.

2 $$P_{ca} = \, 112.7sin\left( {O_{sL} - \pi } \right) + \, 3.1\left( {O_{sL} - 10} \right)^{2} - 95.73$$

where *P*_*ca*_ is the probability of ore pass arching, %; *O*_*sL*_ is the mean ore particle size, cm.

**Figure 5:**
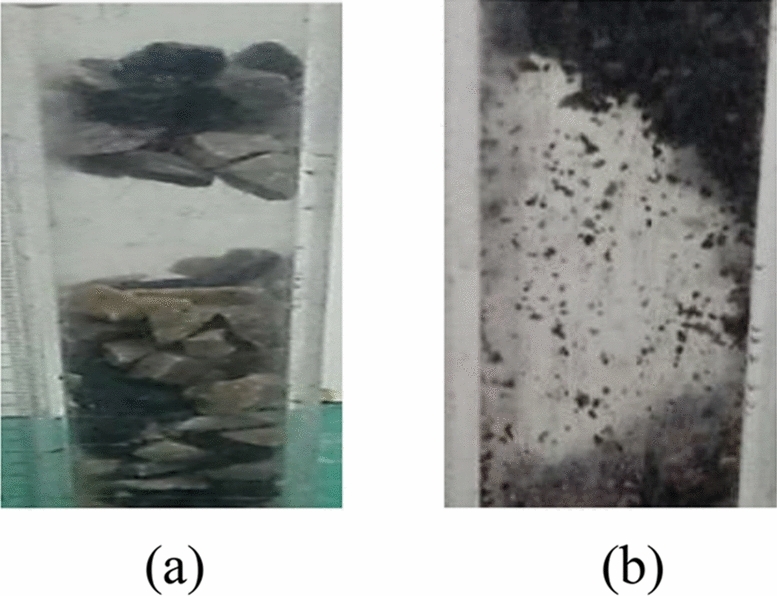
Typical occlusal and bonded arches. (**a**) Occlusal arch, (**b**) bonded arch.

Figure 4 shows that when the ore particle size is overly small, the contact area between the ore particles increases, the bulk structure becomes denser, the bonding effect between the particles is enhanced, and the ore bulk easily forms a bonded arch^16^; when the ore particle size increases, ore extrusion and collision occur during movement, and when the ore bulk arrangement structure is reasonable and the wall of the ore pass is sufficient to support the gravity of the overlying ore, the ore bulk easily forms a bite arch^16^. A typical bite arch and bonded arch are shown in Fig. 5. Therefore, after the increase in the ore grain size of the ore pass arch form from the grain sizes of the bonded arch and bite arch, the ore grains of sizes from 2 to 4 mm exhibit no ore pass arch phenomenon when the bonding and bite effect are not strong.

**Figure 6:**
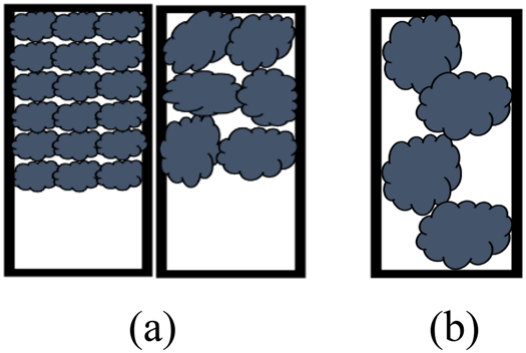
Presence of ore with a ratio of ore grain size to ore pass section that is higher than 30%. (**a**) Form of occlusal arch action, (**b**) large grain size ore in the ore pass.

The ore grain size under the influence of the ore pass arch probability equation (Eq. 2) reflects the ore grain size and the ore pass arch probability of the complex relationship between trigonometric and second-order functions; however, the function image (Fig. 4) does not accurately reflect the complex functional relationship. Although the influence of ore grain size and the ore pass arch probability equation is only applicable to ore grain sizes less than or equal to 10 mm, the calculation of the ratio of ore grain size and ore pass section length shows that for an ore grain size of 8 mm, the ratio of ore grain size and ore pass section reaches 27%. Afterward, with the increase in ore grain size, the ore pass arch probability decreases. As the maximum ore particle size of the ore used in this experiment is 10 mm, the highest ratio of the ore pass section is only 30%. A development with a 30% or greater ratio of ore grain size to ore pass section appears in the form of an occlusion arch (Fig. 6a). If the ratio of ore grain size to the length of the ore pass section is overly high, the phenomenon of Fig. 6b appears; then, the occlusal arch does not form easily, and the probability of the ore pass arch decreases.

**Figure 7:**
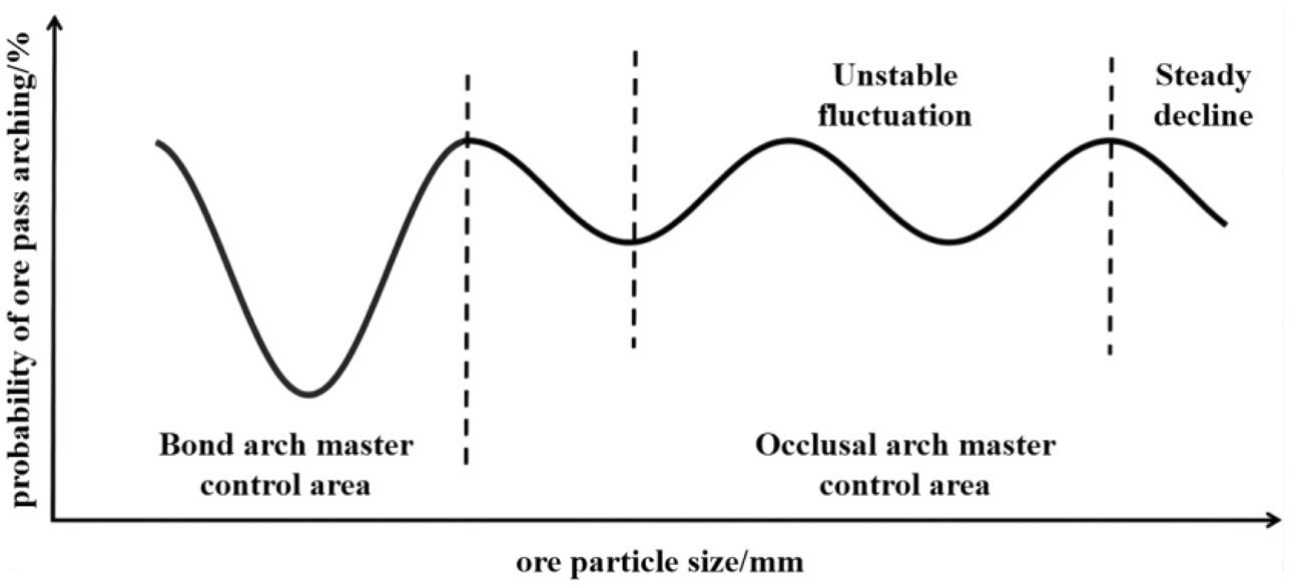
Correlation between the ore grain size and the probability of ore pass arching.

In summary, there may be a development trend in Fig. 7 between the ore grain size and the probability of a nibbling arch in the ore pass. The occlusion form of the ore arch exists in two cases, as shown in Fig. 6a. With increasing ore grain size, the probability of forming an occlusion arch appears to fluctuate. When the phenomenon of Fig. 6b appears, with increasing ore grain size, the occlusal arch does not easily form, and the probability of an ore pass arch decreases stably. Combined with the fitting image and experimental results, it can be seen that the bond arch mainly controls the arching of the ore pass when the ore particle size is in the range of 0–2 mm. With the increase of ore particle size, the interlocking arch mainly controls the arching of the ore pass.

#### Effect of ore pass inclination on the probability of ore pass arching

**Figure 8:**
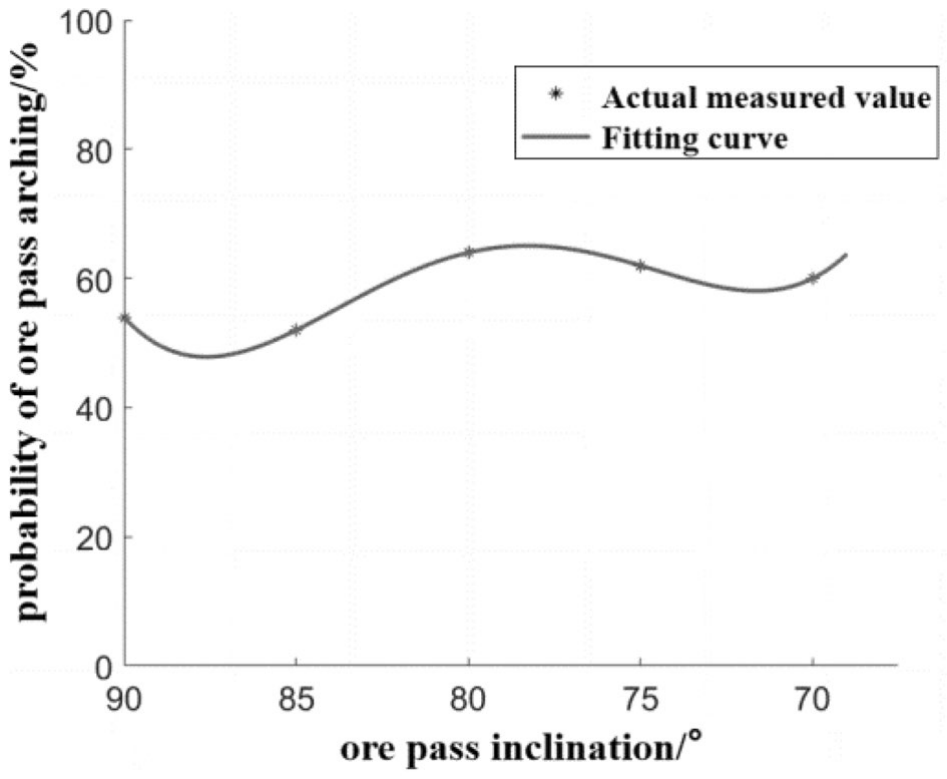
Effect of ore pass inclination on the probability of ore pass arch formation.

The probability of the ore pass under different ore pass inclination is fitted, and the fitted image of the influence of ore pass inclination on the probability of the ore pass arch is shown in Fig. 8.

The fitted R^2^ value is 1, and the fitting effect is excellent. The fitted equation for the probability of the ore pass arch under the influence of the ore pass inclination is shown in Eq. (3), which is applicable to the ore pass inclination in the range of 70°–90°.

3 $$P_{ca} = \, 1.75C_{aL}^{4} - 22.17C_{aL}^{3} + \, 96.25C_{aL}^{2} - 161.8C_{aL} + \, 140$$

where *P*_*ca*_ is the probability of ore pass arching, %; *C*_*aL*_ is the ore pass inclination, °.

The ore pass angle on the ore pass arch probability equation (Eq. 3) reflects that the ore pass angle and the ore pass arch probability show a weak fluctuation relationship. There is a significant 4th-order function relationship, with the ore pass inclination angle decreasing the ore pass arch probability with no significant upward trend. This result shows that the change in the skid angle moderately impacts the skid arch probability. Due to the change in the inclination of the ore pass, the ore bulk flow stress field, arch stress field, energy change, and the combined effect of the three cause the ore pass inclination and arch probability to show a weak fluctuation relationship^37^. When the inclination of the ore pass is near or below the natural resting angle of the ore bulk, the ore bulk is in a stacked state in the ore pass and no longer flows^17^. The natural resting angle of the ore in this experiment is in the range of 25°–42°; thus, there is no increase in the probability of arching due to accumulation.

#### Influence of slip height on the probability of arching in the ore pass

**Figure 9:**
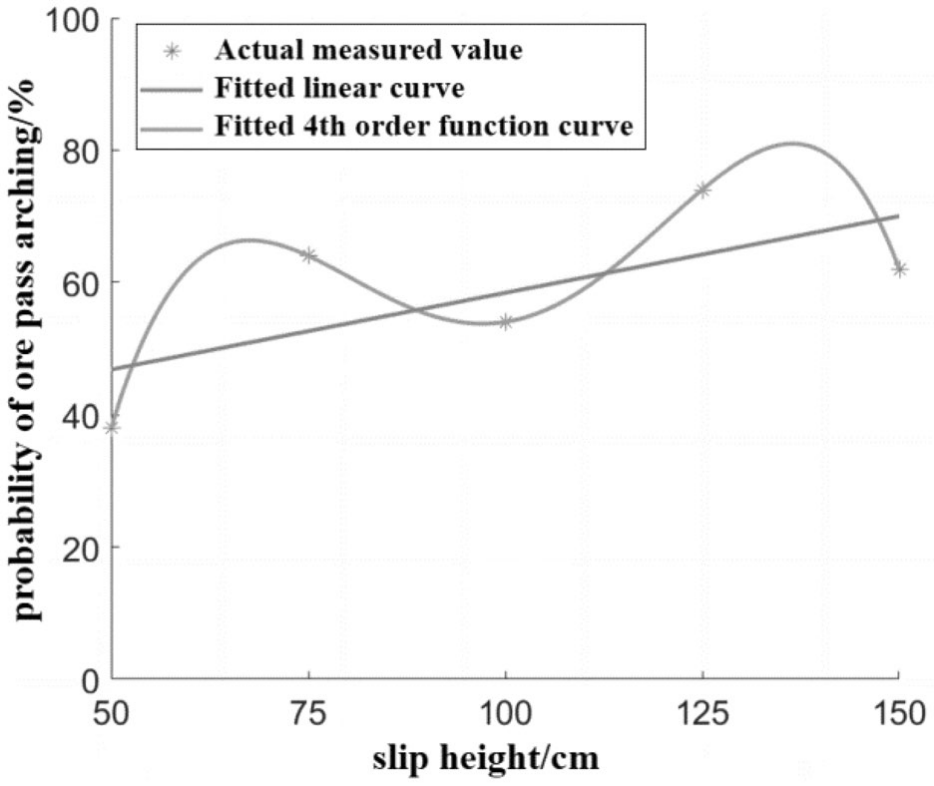
Influence of slip height on the probability of skid arch formation.

The probability of the ore pass under different slip height is fitted, and the fitted image of the influence of slip height on the probability of the ore pass arch is shown in Fig. 9.

The fitted 4th-order function (Eq. 4) and linear function (Eq. 5) reflect the 4th-order polynomial relationship between the slip height and ore pass arch probability, and an overall positive correlation exists, as shown in Fig. 9. The fitted residual mode of the 4th-order function is 4.273 × 10^–12^, indicating an excellent fit; the fitted residual mode of the linear function is 19.66, which is much higher than the fitted residual mode of the 4th-order function while remaining in an acceptable range.

4 $$P_{ca} = - 5.3333M_{h}^{4} + 64.333M_{h}^{3} - 270.67M_{h}^{2} + 467.67M_{h} - 218$$

5 $$P_{ca} = \, 5.8M_{h} + \, 41$$

where *P*_*ca*_ is the probability of ore pass arching, %; *M*_*h*_ is the slip height, cm.

The construction height of the ore pass directly affects the slip height of the ore pass section storage ore; the slip height increases, the impact energy continues to increase, the gravitational potential energy and self-gravity continue to increase, the density increases, and the probability of a knotted arch increases. Of the impact of slip height on the ore pass arch probability appears to have weak fluctuations, which may occur due to the increase in lateral stress and the change in the stress field on the arch probability impact.

### Analysis of the influences of compound factors

#### Influence of ore pass structure on the probability of ore pass arching

**Figure 10:**
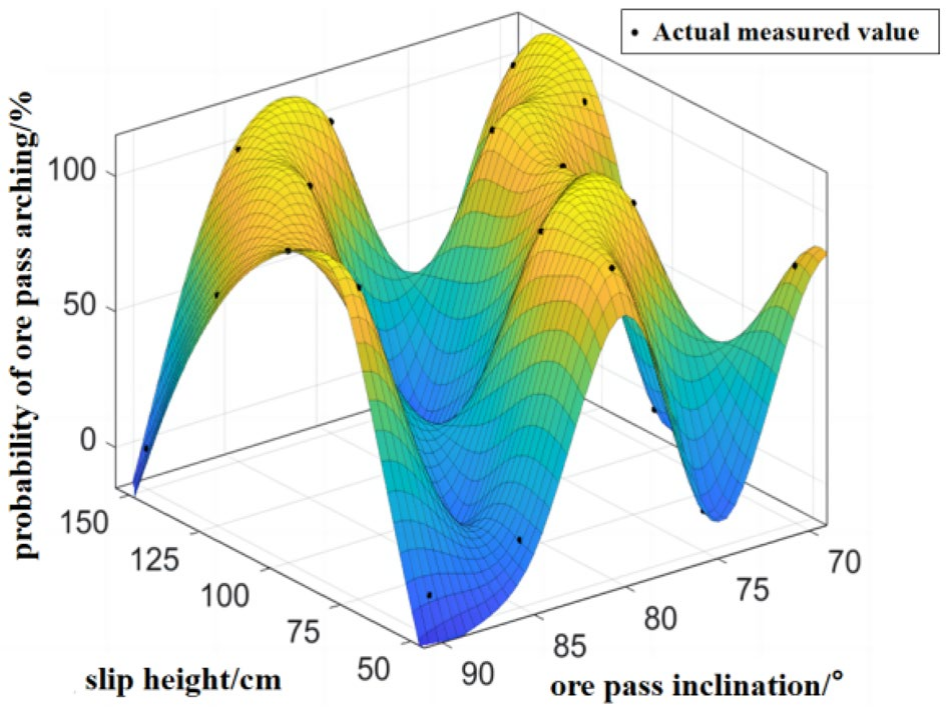
Compound effect of slip height and ore pass inclination on the probability of arch formation in the ore pass.

The probability of arch formation in the ore pass has been adjusted for different slip heights and ore pass inclination angles see Fig. 10.

The image of the fitting function in the graph shows the form of the wave crest and trough. When the ore pass inclination and height increase or decrease simultaneously or a single factor increases or decreases, the ore pass arch probability shows a trend of first increasing and then decreasing. In actual production, the ore pass inclination cannot be adjusted, and the ore slip height can be increased to reduce the ore pass arch probability.

#### Influences of ore properties on the ore pass arch probability

**Figure 11:**
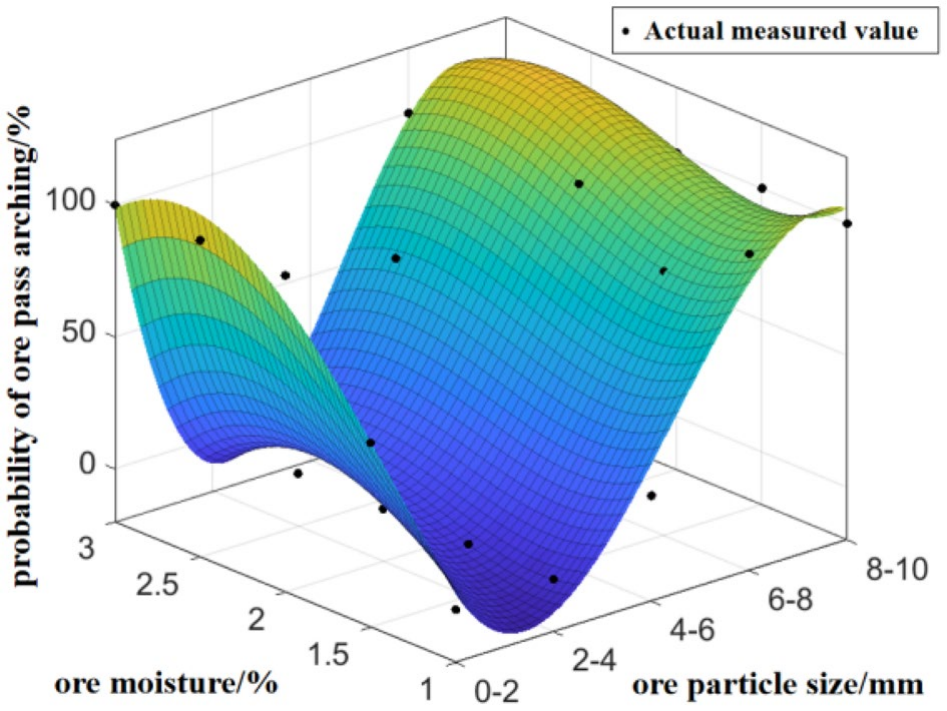
Compound effect of ore moisture and ore particle size on the probability of arch formation in the ore pass.

The probability of arch formation in the ore pass has been adjusted for different ore moisture and ore particle size see Fig. 11.

The effect of ore particle size on the probability of ore pass arching is more significant than that of ore moisture, and the degree of influence of ore moisture on the probability of ore pass arching is not significant due to the ore particle size. High-humidity, large-particle size areas and high-humidity, small-particle size areas both have high probabilities of ore pass arching. The reduction in humidity reduces the high probability of ore pass arching caused by a large particle size.

#### Multifactor slip lining arch probability equation and test

Twelve new variables (*Os*^2^, *Os*^3^, etc.) are generated, and a multivariate nonlinear regression is performed on the variables (a cubic term model is chosen, with a good fit R^2^ of 0.89). The multivariate nonlinear equation for the probability of a kinked arch in the ore pass is obtained as Eq. (6). Multiple linear regression of the experimental sample (the average p value of each parameter is 0.0997, which is less than 0.1, and the regression effect is more significant), and the variation equation of the multiple linear regression of the probability of ore pass arch is obtained, as in Eq. (7).

6 $$\begin{gathered} P_{ca} = - 4766.127 - 130.098O_{s} + 28.741O_{s}^{2} - 1.688O_{s}^{3} - 19690.46M_{c} \\ + 1251428M_{c}^{2} - 2.27 + 07M_{c}^{3} + 184.367C_{a} - 2.28C_{a}^{2} + 0.009C_{a}^{3} \\ + 1.878M_{h} - 0.012M_{h}^{2} + 0.00002M_{h}^{3} \\ \end{gathered}$$

7 $$P_{ca} = 7.8O_{s} + 1240M_{c} - 0.44C_{a} + 0.232M_{h} + 6.6$$

where *P*_ca_ is the probability of ore pass arching, %; *O*_s_ is the mean ore particle size, cm; *M*_c_ is the ore moisture, %; *C*_a_ is the ore pass inclination, °; and *M*_h_ is the slip height, cm.

The application ranges of the multivariate nonlinear regression equation and multivariate linear regression equation for the ore pass arch probability are as follows: the ore moisture content is between 1 and 3%, the ratio of ore grain size to ore pass section size is controlled at 1/3, the slip height is between 50 and 150 m, and the ore pass inclination angle is between 70° and 90°.

The values of the change in the probability of the slip arch for the fitted and experimental samples are calculated using Eqs. (6) and (7), and the absolute error values are calculated. The data test results are shown in the Table 6. The mean value of the cumulative absolute error of the multiple nonlinear regression equation is 11.906, which is within the acceptable range. The mean value of the cumulative absolute error of the multiple linear regression equation is 30.736, which is larger. Therefore, the multivariate nonlinear regression equation is used as the equation of the ore pass knotted arch.

**Table 6:** Error analysis of fitting.

Experimental number	Measured value	Nonlinear regression prediction value	Error magnitude (nonlinear)	Linear regression predicted value	Error magnitude (linear)
1	0	32.82857	−32.8286	−1.2	−1.2
2	0	−23.4572	23.45715	22.4	22.4
3	20	25.11428	−5.11428	46	26
4	100	92.54285	7.457145	69.6	−30.4
5	100	92.82857	7.171431	93.2	−6.8
6	50	63.11429	−13.1143	18.8	−31.2
7	0	−3.31428	3.314278	42.4	42.4
8	10	32.11429	−22.1143	66	56
9	80	64.82858	15.17142	60.6	−19.4
10	100	83.82858	16.17142	73.2	−26.8
11	100	83.6857	16.3143	38.8	−61.2
12	0	−10.4572	10.45715	33.4	33.4
13	20	35.6857	−15.6857	57	37
14	100	99.6857	0.314297	69.6	−30.4
15	100	110.5428	−10.5429	93.2	−6.8
16	100	90.82858	9.171421	29.8	−70.2
17	0	19.97144	−19.9714	53.4	53.4
18	70	49.97144	20.02856	66	−4
19	100	109.8286	−9.82858	89.6	−10.4
20	100	99.97144	0.028564	84.2	−15.8
21	100	83.68571	16.31429	49.8	−50.2
22	0	0.685713	−0.68571	62.4	62.4
23	10	11.97143	−1.97143	57	47
24	100	96.54285	3.457145	80.6	−19.4
25	100	116.9714	−16.9714	104.2	4.2

## Ore pass arch location characteristics

### Description statistics of the location of the ore pass arch

**Figure 12:**
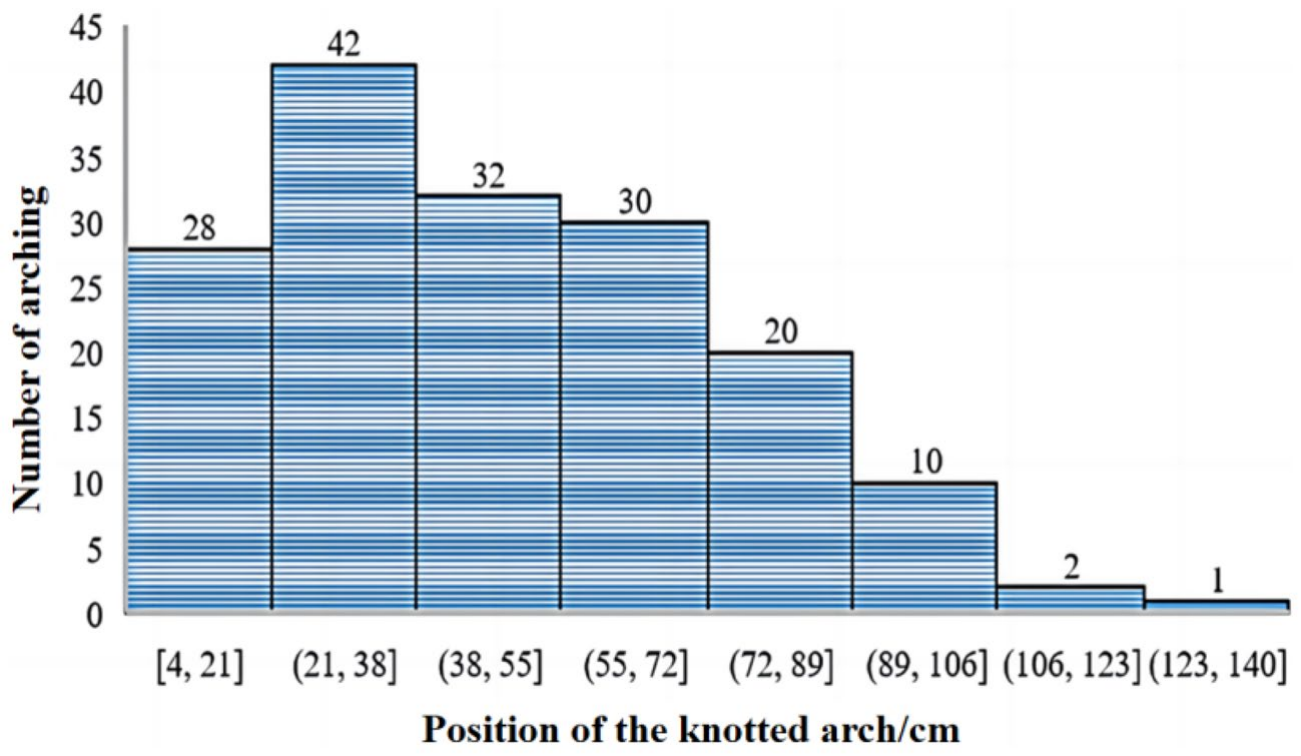
Cumulative arch position statistics.

According to the statistics of the experimental results, the cumulative number of arching positions was 165, and the number of arching appearances in each interval was described by 17 cm as the interval. See the statistical chart of cumulative archival positions (Fig. 12), in which the number of archival positions in the interval (21 cm, 38 cm) was as high as 42, the cumulative number of archival appearances in the interval (4 cm, 72 cm) was as high as 132, accounting for 80% of the total number of archival positions. The cumulative number of archiving times in the (106 cm, 140 cm) interval was only 3, and the cumulative percentage was only 1.8%.

### Analysis of the position of the ore pass arch under different slip heights

**Figure 13:**
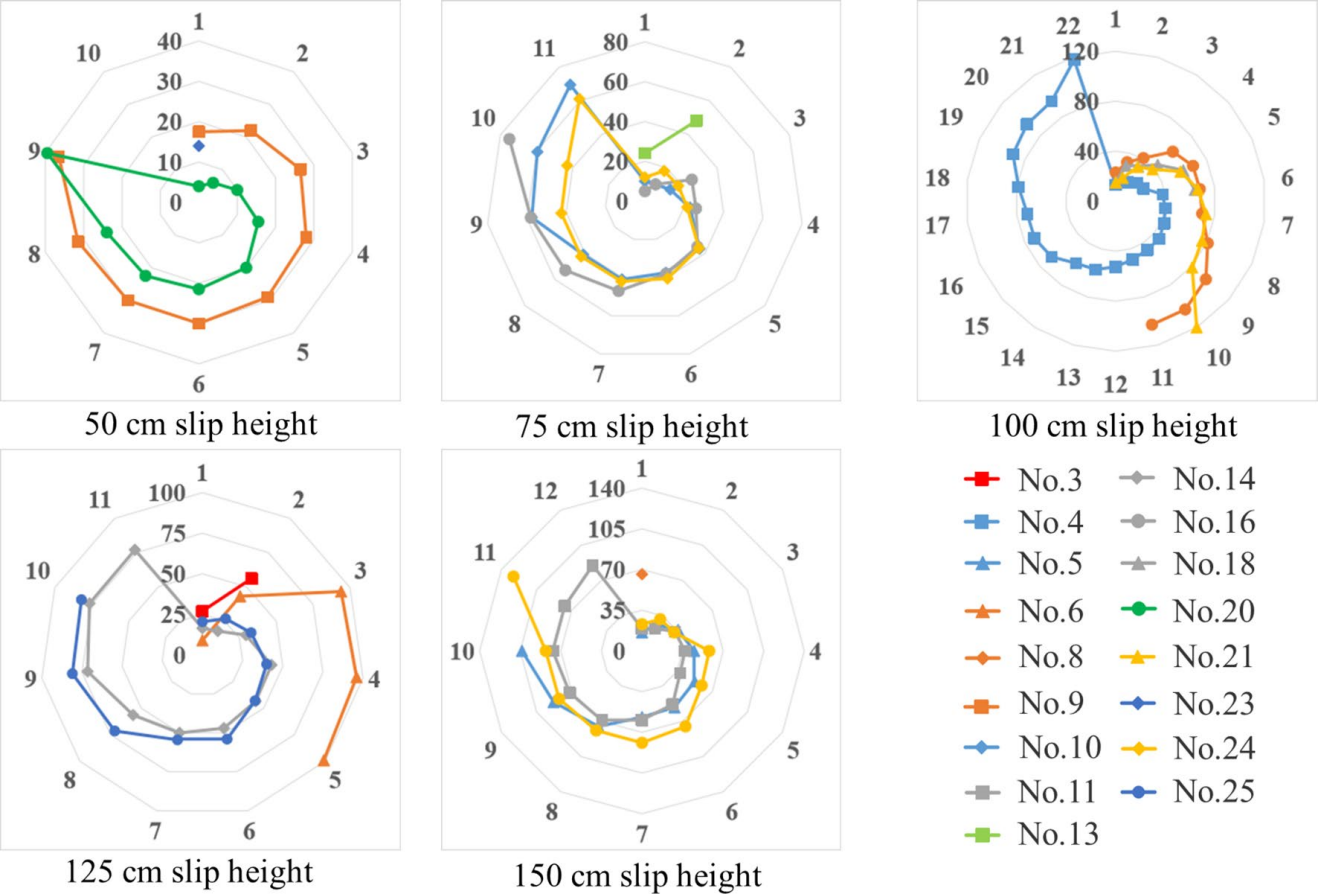
Knot arch position information map for each slip height.

In the actual project, the initial value of the skid height during the ore pass release directly affects the position of the knotted arch of the ore pass release. The distribution of the arch position is observed separately for ore pass heights of 50 cm, 75 cm, 100 cm, 125 cm and 150 cm (see Fig. 13). The parameter values of the corresponding factors for each experiment are shown in Table 5.

**Figure 14:**
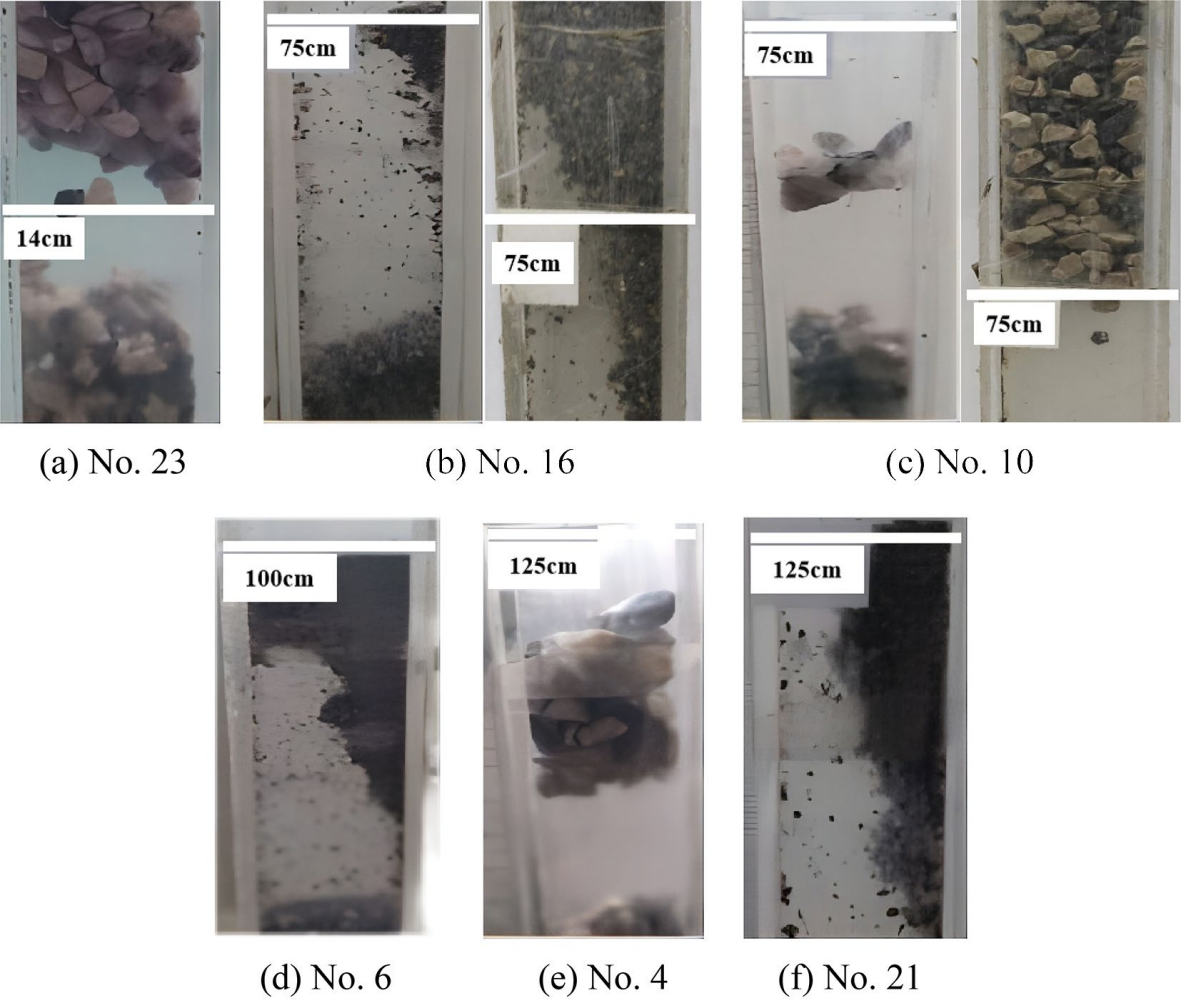
Arching phenomenon at each skimming height.

The arching times of experiment numbers 1, 2, 7, 12, 17 and 22 are 0; thus, there is no corresponding arching location information. An observation of 50 cm slip height experimental ore particle size shows that the particle size of 0–4 mm does not have apparent arch phenomenon; the arch phenomenon is concentrated in the particle size of 6–10 mm when the arch form is that of an occlusion arch. Under the particle size of 4–6 mm, the arch form is a combination of ore bonding and occlusion force, and the bonding effect is larger (see Fig. 14a).

Experiments 16 and 10 both show the phenomenon of a knotted arch near the initial value of 75 cm of the skimming height, as shown in Fig. 14b, c, when the ore bonding and biting action are extremely strong. By observing the other parameters of experiments 10, 16 and 24, it is found that the stronger the bonding is or the greater the occlusion of the ore is, the higher or lower the development of the skid arch location, respectively. In experiment 13, there is a combined occlusion-bonding effect; however, the bonding and occlusion of the ore are not strong, and the density is not high; thus, the position of the arch is in the middle and the amount of arching is low.

In experiment 6, the arching phenomenon occurs near the initial value of 100 cm of the skimming height, as shown in Fig. 14d; the ore bonding is extremely strong at this time. The significant occlusion effect does not appear near the initial value of the slip height arch phenomenon. Unlike the characteristics of the location of the arch in experiment 10 at 75 cm slip height, the change in ore moisture and slip height prompts no occlusal arch near the initial value of 100 cm slip height.

The highest arch locations of experiments 4 and 21 are 118 cm and 120 cm, respectively (see Fig. 14e, f), where the occlusion and bonding action prompt the occurrence of arching near the initial value of the slip height; this phenomenon is more similar to the arch location characteristics of experiment 10 at a 75 cm slip height. Experiment 18 has the fewest arches in the center of the arch location.

At a slip height of 150 cm, no arching occurs near the initial value of the slip height. Experiment 8 has the fewest centered arches. The arching phenomenon only occurs twice in the arching position of 100–150 cm. The arching position of experiment 19 is higher than that of experiment 11. The improvement in the arch position by occlusion between the ores is more significant than that by bonding. Although the particle size of experiment 5 is 8–10 mm, the change in the ore pass dip angle and the ore moisture content decreases the occlusion effect.

### Slip arch interval zoning

**Figure 15:**
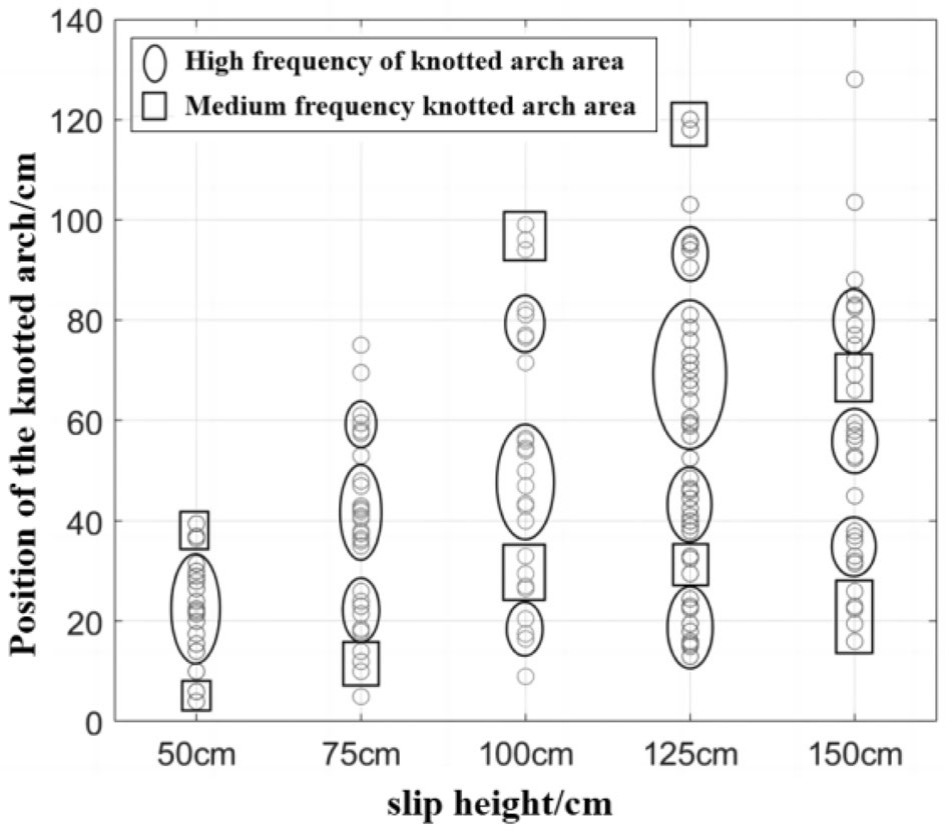
Description of the interval zone of the ore pass arch under different skidding heights.

The location of the ore pass arch is divided into high-frequency, medium-frequency and low-frequency arch regions. The knot arch high-, medium- and low-frequency division principle is as follows: the average value of the difference between the knot arch position in the high-frequency knot arch region is less than 1.5, the average value of the difference between the knot arch position in the medium-frequency knot arch region is greater than or equal to 1.5 and less than 3, and the average value of the difference between the knot arch position is greater than 3 regions for the low-frequency knot arch region. Different slip heights under the ore pass arch interval zoning description are shown in Fig. 15.

Combined with the analysis of the slip height of the ore pass arching interval description map, experiments 9, 20, 10, 24, 25, 5, 11 and 19 appear to be arching location concentration areas; other arching location experiments are more scattered. In the direction of the coordinate axis, only one arching phenomenon occurs in experiment 6 after experiment 3; the significant increase in the slip height effectively reduces the arching phenomenon near the release port. The high-frequency and medium-frequency arching areas are not continuous, and the high-frequency and medium-frequency arching areas do not increase with increasing slip height. The highest high-frequency arching area shows a normal distribution with increasing slip height; the high-frequency arching area reaches its highest value at a slip height of 125 cm, and this area is [90.5 cm, 95.5 cm].

## Conclusions

1. The influence of ore moisture on the arching of the ore pass is mainly that the existence of free water and bound water affects the adhesion of ore particles, the influence of ore particle size on the arching of the ore pass, and the existence of ore particles and the bite force between the ore pass wall. The influence of the ore pass dip angle on the arching of the ore pass is due to the change of the ore pass dip angle affecting the change of the ore pass stress field. With the increase of ore moisture, the adhesion of ore particles first increases and then decreases. With the increase of ore particle size, the adhesion of ore particles decreases first, then increases and then decreases. When the ore moisture in the ore pass does not reach the saturation value of the bound water and the fluidity is good, the ore is normally discharged. If the ore humidity in the ore pass exceeds the saturation value of the bound water, the ore humidity (e.g., water injection into the ore pass, etc.) is increased to reduce the probability of arching.
2. When the ore pass inclination and height increase or decrease simultaneously or a single factor increases or decreases, the ore pass arch probability shows a trend of first increasing and then decreasing. The ore pass inclination angle and arch probability show a weak trigonometric relationship, and there is a significant 4th-order function relationship; the ore pass inclination angle decreases the ore pass arch probability with no significant upward trend. The change in the inclination angle of the ore pass more moderately affects the probability of the ore pass arch. The slip height and arch probability have a 4th-order polynomial relationship and have a positive correlation. When the angle of inclination of the ore pass is close to or less than the natural angle of repose of the ore rock bulk, the ore rock bulk will be in the ore pass in the accumulation state and will no longer flow. In order to reduce the accumulation of ore caused by the chute arch phenomenon, the angle of inclination of the chute should be greater than the natural angle of repose of the ore rock bulk. When constructing the shaft, the inclination angle of the shaft should be controlled at the best slightly inclined angle of the straight shaft.
3. The degree of influence of ore moisture on the probability of arching in the ore pass is not significant due to the ore particle size. High-humidity, large-particle size areas and high-humidity, small-particle size areas both have high probabilities of ore pass arching. Ore moisture and the probability of ore pass arching show a quadratic relationship. Before the ore moisture reaches the saturated water content, the combined water dominates the ore pass arching probability; after reaching the saturated water content, the free water dominates the ore pass arching probability. With the increase in the ore particle size, the arch form of the ore passes changes from the bonded arch to the occluded arch. The ore particle size and the arch probability of the skid present a complex relationship between trigonometric and second-order functions. The ore particle size reaches a certain range before the bonded arch main control skid arch factors; the increase in ore particle size affects the bite arch, which mainly controls the skid arch probability.
4. The arching phenomenon easily occurs near the ore release opening when the height of the ore is low. Strong occlusion and bonding promote the arching phenomenon near the initial value of the slip height. When the bonding and occlusion of the ore are not strong and the density is low, the arching phenomenon does not easily occur in the middle of the ore pass. At a 150 cm slip height, the occlusion between the ore on the arching position is more significant than the bonding on the arching position. The high-frequency and medium-frequency arching areas are not continuous, and the high-frequency and medium-frequency arching areas do not increase with increasing slip height. The highest high-frequency arching area shows a normal distribution with increasing slip height; the highest high-frequency arching area is reached at a slip height of 125 cm, and this area is [90.5 cm, 95.5 cm]. In the process of drawing ore in low ore passes, the height of ore passes should be maintained below 50 m. In addition, in the process of drawing ore in high and deep ore passes, the height of ore passes should be maintained at 100 m or 150 m.

## Data Availability

The datasets generated or analyzed during the current study are not publicly available due the data set is being used for the next study but are available from the corresponding author on reasonable request.
